# Analysis of Different Post-Operative Hyperamylasemia Criteria for Defining Post-Pancreatectomy Acute Pancreatitis After Distal Pancreatectomy—A Retrospective Single-Center Study

**DOI:** 10.3390/jcm15051803

**Published:** 2026-02-27

**Authors:** Lukas Heinrich Poelsler, Ruben Bellotti, Daniel Pably, Dagmar Morell-Hofert, Eva Maier, Benno Cardini, Rupert Oberhuber, Thomas Resch, Florian Ponholzer, Felix J. Krendl, Christian Margreiter, Stefan Schneeberger, Dietmar Öfner, Manuel Maglione

**Affiliations:** 1 Department of Visceral, Transplant and Thoracic Surgery, Center of Operative Medicine, Medical University of Innsbruck, 6020 Innsbruck, Austria; lukas.poelsler@i-med.ac.at (L.H.P.); ruben.bellotti@i-med.ac.at (R.B.); daniel.pably@student.i-med.ac.at (D.P.); eva.maier@i-med.ac.at (E.M.); benno.cardini@i-med.ac.at (B.C.); rupert.oberhuber@i-med.ac.at (R.O.); thomas.resch@i-med.ac.at (T.R.); florian.ponholzer@i-med.ac.at (F.P.); felix.krendl@i-med.ac.at (F.J.K.); christian.margreiter@tirol-kliniken.at (C.M.); stefan.schneeberger@i-med.ac.at (S.S.); dietmar.oefner@i-med.ac.at (D.Ö.); 2 Department of Radiology, Medical University of Innsbruck, 6020 Innsbruck, Austria; dagmar.morell@i-med.ac.at

**Keywords:** post-pancreatectomy acute pancreatitis, distal pancreatectomy, clinically relevant POPF, postoperative hyperamylasemia, complications

## Abstract

**Background/Objectives**: The International Study Group for Pancreatic Surgery has recently defined post-pancreatectomy acute pancreatitis (PPAP), stating that sustained postoperative hyperamylasemia (POH) for at least 48 h is a pivotal criterion. However, the clinical relevance of POH and PPAP following distal pancreatectomy remains uncertain. This study compares two PPAP definitions differing in POH criteria. **Methods**: We retrospectively analyzed all patients who consecutively underwent distal pancreatectomy at our institution (2010–2023). PPAP diagnosis required clinical symptoms, characteristic CT findings, and either sustained POH ≥ 48 h (standard group) or transient POH less than 48 h (modified group). Outcomes were compared between definitions. **Results**: Among 207 patients included, in the standard group, PPAP was diagnosed in 12 (5.8%), and in the modified group in 27 (13.0%) patients. Independent of the applied POH criteria, PPAP was associated with the occurrence of clinically relevant postoperative pancreatic fistulas (standard: 66.7% vs. 23.7%; *p* < 0.001; modified: 44.4% vs. 23.7%; *p* = 0.027). Post-pancreatectomy hemorrhage and major complications (Clavien–Dindo grade ≥ III) were also significantly more frequent in patients with PPAP. This was mirrored by a significantly longer length of stay and higher costs. However, in the standard group, PPAP more often resulted in pancreas-specific and major complications compared to the modified group. Of note, in the standard group, only 50% of patients with POH progressed to PPAP, and one-third of patients suffering from PPAP did not develop harmful sequelae. **Conclusions:** PPAP is an uncommon, however clinically relevant complication following distal pancreatectomy that is better captured using the standard POH definition. Still, further stratification is needed to aid in the prediction of the clinical course.

## 1. Introduction

Following Connor’s systematic literature review, in which he suggested that postoperative pancreatitis should be recognized as a stand-alone pancreas-specific complication following pancreatectomy [1], several groups have concurred with his recommendation. In fact, postoperativepancreatitis was not only associated with an increased incidence of serious pancreatectomy-specific complications, such as clinically relevant postoperative pancreatic fistula and post-pancreatectomy hemorrhage, but also with higher numbers of severe postoperative complications [2,3,4,5,6].

In a non-operative setting, the diagnosis of acute pancreatitis requires at least two out of three criteria, including: abdominal pain, a three-fold elevation of pancreatic enzymes in the serum, and typical radiological findings on cross-sectional imaging [7]. However, in patients undergoing pancreatectomy, the diagnosis may be hampered by postoperative pain and analgesics, as well as by an impaired enzyme release into the blood/serum due to the partial loss of glandular tissue, chemotherapy-associated fibrosis, and obstructive pancreatitis, eventually resulting in lower pancreatic enzyme values in the serum [8]. Taking these aspects into account, the International Study Group for Pancreatic Surgery (ISGPS) came up with a consensus where post-pancreatectomy acute pancreatitis (PPAP) was defined as an association of sustained postoperative hyperamylasemia (POH) for at least 48 h accompanied by radiologic alterations consistent with pancreatitis and a clinically relevant deterioration of the patients’ condition during the postoperative course [9].

While the PPAP definition and its relevance are widely accepted and were recently validated for pancreatoduodenectomy [3,10,11], data regarding distal pancreatectomy are limited and discordant. Depending on whether the definitions used rely merely on serum markers or not, the incidences of PPAP following distal pancreatectomy vary between 0.25% and 45% [12,13,14]. While PPAP has been described and validated in a recent paper in the setting of pancreaticoduodenectomy [10] its relevance for distal pancreatectomy, as well as the question of whether the inclusion of cross-sectional imaging might better capture the clinical relevance than POH alone, continues to be a matter of debate.

The aim of this study was to analyze the frequency and the clinical impact of PPAP following distal pancreatectomy at our center. Considering the debate regarding the relevance of persistent POH following distal pancreatectomy, the criteria proposed by the ISGPS group were compared to a modified version where transient rather than sustained POH for at least 48 h sufficed to meet the criteria for PPAP.

## 2. Materials and Methods

This is a retrospective analysis of an auditable dataset [15] collected prospectively from all consecutive patients who underwent distal pancreatectomy between 2010 and December 2023 at the department of Visceral, Transplant, and Thoracic Surgery at the Medical University of Innsbruck, Austria. Both STROBE guidelines [16] as well as the Declaration of Helsinki [17] were adhered to in this study. The study was approved by the ethics committee of the Medical University Innsbruck (registry number: 1118/2024), and patient consent was waived according to the ethics committee’s statement because of its retrospective character.

### 2.1. Data Collection and Cohort Characteristics

Regardless of spleen removal, all patients who consecutively underwent distal pancreatectomies were included, primary as well as secondary resections, and those in the context of extended resections. None of these patients underwent distal pancreatectomy as surgical treatment for acute pancreatitis. PPAP occurrence was defined according to the ISGPS definition regarding alteration of the pancreatic remnant consistent with pancreatitis on postoperative CT scan and clinically relevant changes in patient management [9]. However, concerning POH, two different definitions were applied: (1) patients with sustained POH for at least 48 h (standard group) and (2) patients with transient POH for less than 48 h (modified group). POH was defined as amylasemia higher than the institutional upper limit (53 U/L).

Furthermore, post-pancreatectomy specific complications, such as clinically relevant pancreatic fistula and post-pancreatectomy hemorrhage, were captured according to the ISGPS definition [18,19]. Other data, including major postoperative complications classified as Clavien–Dindo ≥ III, [20] hospital readmission and relaparotomy rates, length of stay, and 90-day mortality were also analyzed. An analysis of the overall costs (expressed in euros, €) was also performed. The distribution of costs was as follows: operative costs accounted for 30.84%, 52.55% was allocated to inpatient care, and 16.61% was designated for additional intensive care unit stays. Data were provided by the financial department of the Medical University of Innsbruck.

**Figure 1:**
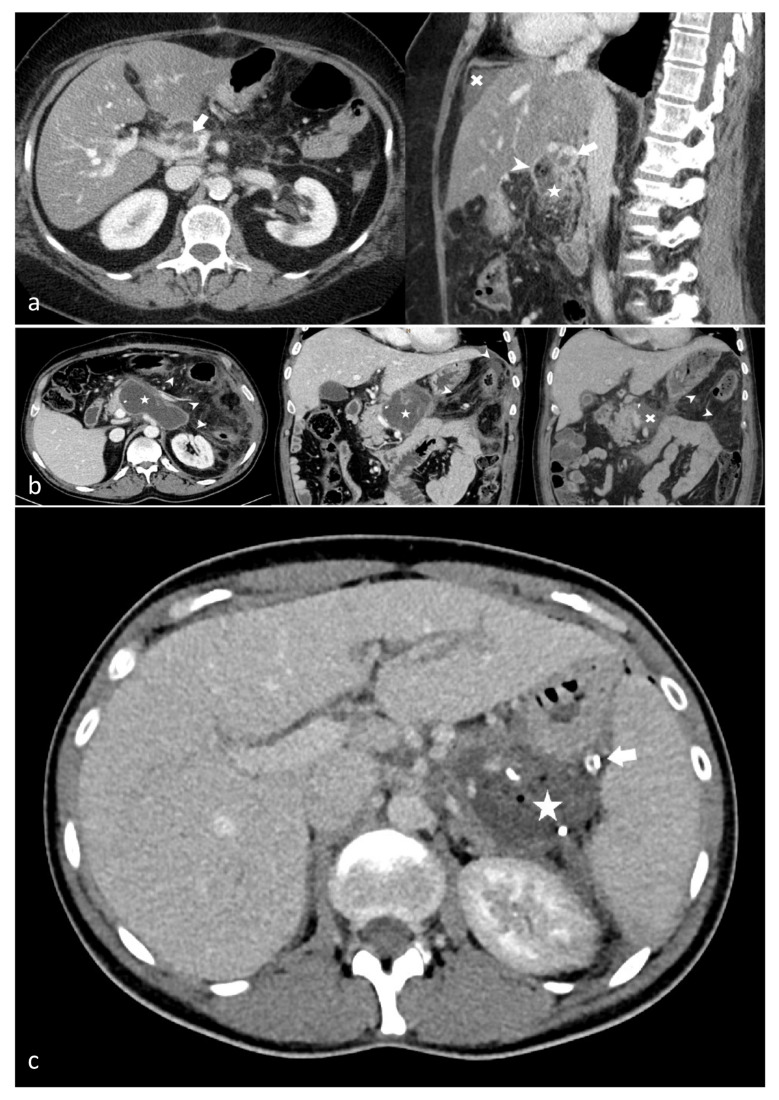
**(a–c). Exemplary findings in PPAP scans:** (**a**) Arrow: Portal vein thrombosis in the CT scan on 10th POD; Arrowhead: Drainage; Star: Remnant pancreas; Cross: Perihepatic ascites; (**b**) Star: Pseudocyst in the CT scan several weeks post-operation; Arrowheads: Mesenteric fat stranding; Cross: Peripancreatic fluid collection in the first postoperative CT scan; (**c**) Star: Air-containing peripancreatic collection in the first postoperative CT scan; Arrow: Drainage.

All radiological findings were retrospectively reevaluated by an experienced radiologist. Within all scans, we screened for the following signs of (sub-)acute pancreatitis: peripancreatic fat stranding, peripancreatic fluid collections (acute peripancreatic fluid collection, acute necrotic collection, walled-off pancreatic necrosis, parenchymal necrosis, and other (post-)inflammatory complications (pseudocysts, pseudoaneurysms, portal vein thrombosis [21]. Exemplary pictures demonstrating radiological findings characteristic of PPAP are provided in Figure 1a–c. Radiological findings, especially peripancreatic fluid collections and imbibition of mesenterial fat, are commonly seen in patients after pancreas resections [22] and therefore were interpreted alongside the clinical state of the patient.

The study’s main endpoints were the frequency and the clinical impact of PPAP depending on the different POH definitions considered (standard vs. modified group). The other endpoint was the economic implications of this condition. Those were measured by the billed amount of the hospital stay for our center. This was influenced by the length of stay, length of intensive care unit stay, and additional diagnostic and interventional actions.

### 2.2. Statistical Analysis

Statistical analysis was performed using IBM SPSS Version 27 (SPSS Inc., Chicago, IL, USA). Continuous variables were reported as median (with 25% and 75% percentiles), and categorial variables were described as frequencies and percentages. Fisher’s exact tests were used to compare categorical variables. Two-sided *p*-values ≤ 0.05 were considered statistically significant.

## 3. Results

In total, 207 patients were included in this study. Overall, 104 were male (50.2%) and 103 female (49.8%). The median age was 63 years (51–72). Detailed patient characteristics are shown in Table 1. The most frequent surgical procedure was conventional distal pancreatectomy (n = 158; 76.3%), followed by the radical antegrade modular pancreatosplenectomy procedure (n = 37; 17.9%) and pancreatic tail resection (n = 12; 5.8%). Simultaneous splenectomy was performed in 119 patients (57.5%). Resection of the portomesenteric axis occurred in 13 patients (6.3%). A total of 57 patients (29.2%) underwent laparoscopic surgery.

Among this patient collective, 110 patients (53.1%) suffered from pancreatic malignancies, with adenocarcinomas being the most frequently observed entity (n = 57, 51.8%), followed by neuroendocrine neoplasms (n = 54, 49.2%). Among the other lesions (n = 73; 35.3%), intraductal papillary-mucinous neoplasms and chronic pancreatitis were the most frequent indications for surgery (for more details, see Table 2).

When stratifying patients for PPAP occurrence according to the ISGPS criteria (standard group), 12 patients (5.8%) developed this condition. When comparing demographic and surgery-related data between patients with or without developing PPAP, there were no significant differences (for more details, see Table 3).

Concerning the occurrence of post-pancreatectomy specific complications, these were significantly more frequent in patients with PPAP (clinically relevant postoperative pancreatic fistulas: 66.7% vs. 23.7%; *p* < 0.001; post-pancreatectomy hemorrhage: 41.7% vs. 7.7%; *p* < 0.001, respectively). In addition, the incidence of peripancreatic fluid collections (66.7% vs. 32.9%; *p* = 0.002), pleural effusions (58.3% vs. 21.6%; *p* = 0.005), and major surgical complications (Clavien–Dindo ≥ III) were observed more frequently in patients experiencing PPAP (66.7% vs. 26.1%; *p* < 0.001). In contrast, relaparotomy and readmission rates did not reach statistically significant differences between patients with and without PPAP (33.3% vs. 14.0%; *p* = 0.069 and 33.3% vs. 19.0%; *p* = 0.193, respectively). The most common reason for readmission in both groups was fever due to an infected peripancreatic fluid collection and abdominal pain. Leading causes for relaparotomy were post-pancreatectomy hemorrhage and non-radiologically drainable retentions. Of note, 6.2% of patients experiencing POH did not develop PPAP (for more details, see Table 4).

When stratifying patients with PPAP according to the modified POH definition (modified group), PPAP was observed in 27 patients (13.0%). In this group, patients experiencing PPAP underwent venous resection of the portomesenteric axis (14.8% vs. 5.0%; *p* = 0.027). The other factors did not differ between the two groups (for more details, see Table 5).

Also, when applying the modified definition, patients suffering from PPAP showed higher rates of pancreatectomy-specific complications, like clinically relevant postoperative pancreatic fistulas (44.4% vs. 23.7%; *p* = 0.027) and post-pancreatectomy hemorrhage (25.9% vs. 7.7%; *p* < 0.001). Likewise, peripancreatic fluid collections were associated with PPAP (59.3% vs. 32.9%; *p* = 0.018). Again, major complications classified as Clavien–Dindo ≥ III were more frequently present in patients with PPAP (44.4% vs. 26.1%; *p* = 0.007), and this fact was also reflected in higher relaparotomy rates, although this difference was not statistically significant (14.0% vs. 22.2%; *p* = 0.099). When applying the modified definition, POH was observed in 11.1% of patients not developing PPAP (for more details, see Table 6).

When comparing the postoperative course between the standard and modified group, POH on postoperative day 1 was significantly higher in patients with PPAP according to the ISGPS definition (205 U/L vs. 138 U/L; *p* = 0.026). With regard to postoperative complications, only the incidence of pleural effusions was significantly more frequent in patients suffering from PPAP according to the former group (58.3% vs. 37.0%; *p* = 0.002). Pancreatectomy-specific complications occurred more often when applying the ISGPS definition of PPAP; however, these differences did not reach statistical significance (clinically relevant postoperativepancreatic fistulas: ISGPS 66.7% vs. modified 44.4%; *p* = 0.053, and post-pancreatectomy hemorrhage: standard 41.7% vs. modified group 25.9%; *p* = 0.058). All other clinical outcomes had a similar distribution within the two groups (for more details, see Table 7).

The median length of stay was 12 days (8–19). In patients having PPAP, the length of stay was significantly prolonged, 27 days (11–47) in the standard group (*p* = 0.002); 14 days (10–28) in the modified group (*p* = 0.014). There was no difference in length of stay when comparing patients with PPAP according to the two different definitions.

The average costs per hospital stay per patient were €13.231 (11,119–16,293). The overall costs were significantly higher in patients with PPAP regardless of the definition (standard group €15.116 (10,378–51182) vs. €13.231 (11,129–16,285); *p* = 0.002; modified group €13.537 (9992–20,439) vs. €13.231 (8996–16,163); *p* = 0.041). When comparing costs between the two different definitions, no significant difference could be observed (*p* = 0.132).

## 4. Discussion

This study shows that PPAP after distal pancreatectomy is clearly associated with a significantly higher complication burden. In particular, we showed that independently from the type of POH criteria applied, PPAP after distal pancreatectomy implicates a higher occurrence of clinically relevant postoperative pancreatic fistulas, post-pancreatectomy hemorrhage, severe complications (Clavien–Dindo ≥ III), and longer length of stay. This correlation was also reflected by higher costs concerning the hospital stay.

In general, the observation that PPAP is less likely to occur following distal pancreatectomy might be related to the minor mobilization of the gland compared to pancreaticoduodenectomy. In fact, increased physical pressure, such as that experienced during surgery, can induce acute pancreatitis [23]. However, the heterogeneous definitions applied in the literature, which encompass procedures ranging from pancreatic tail resections to extended left pancreatic resections, might also challenge this observation [24].

Of note, the stump closure technique does not seem to affect the incidence of PPAP [25,26].

The incidence of PPAP reported in our study is in line with incidence rates reported in the literature. In studies considering only serological parameters for PPAP diagnosis, an incidence varying between 25% and 45% was observed [12,25]. In contrast, analyses that also included morphologic criteria (either pancreatitis-specific CT or intraoperative findings) showed a PPAP occurrence of approximately 2% [13,27]. The incidence in our cohort reflects more than that of the latter.

In addition, we observed that compared to our results, the occurrence of severe postoperative complications was lower for studies considering only serology as a diagnostic criterion (18.4%) [12,25]. However, when considering studies that also included morphologic criteria, incidences of severe postoperative complications were similar to our study (45%) [13,27]. This supports the consensus definition of the ISGPS, which includes mandatory imaging to diagnose PPAP [9] as it better identifies patients who will develop clinically relevant complications. However, the timepoint at which imaging should be performed continues to be a matter of debate. The similar incidence of postoperative complications paired with a more than two times higher rate of diagnosed PPAP cases compared to the Heidelberg [13] and the Karolinska group [27], who both applied morphologic criteria, suggests that our center has a lower threshold for performing CT scans.

When considering that PPAP might represent the earliest event in a severe complication cascade, recognizing it as early as possible could help mitigate its consequences. Within this context, an interesting approach is presented by the recently published PORSCH trial [28]. The implementation of a structured postoperative approach regarding drain removal, antibiotic treatment, and indication for CT scan resulted in a significant reduction in a composite outcome parameter, including post-pancreatectomy hemorrhage requiring interventions, organ failure, and death. Such standardized approaches have the potential to facilitate more effective identification of patients at risk of developing PPAP. This is particularly salient given the probable shared pathophysiology underpinning the occurrence of clinically relevant postoperative pancreatic fistulas and PPAP, and the potential for post-pancreatectomy hemorrhage to emerge as a significant consequence of clinically relevant postoperative pancreatic fistulas or PPAP [29,30].

The clinical relevance of overall and pancreas-specific complications is also mirrored by the costs of the hospital stay. We and others have already observed that PPAP following pancreaticoduodenectomy results not only in a prolonged length of stay but also in higher costs [3,11,27,31]. The same observation was made in this study. The higher complication burden of patients diagnosed with PPAP corresponded to a longer length of stay and higher cost burden.

In this study, the incidence of readmissions did not reflect the difference in clinically relevant complications between patients who developed PPAP and those who did not. This may be due to the small sample size, as well as the generally high readmission rate in this patient cohort.

When comparing our two study groups, implementing a sustained POH for at least 48 h more accurately reflects the clinical course of patients developing PPAP. Although not reaching statistical significance, applying the standard definition resulted in a higher number of patients developing severe, pancreatectomy-specific complications compared to the modified definition.

POH following pancreatic resections is frequently observed. Its potential role in preceding severe complications is well described; however, most publications have focused on patients receiving resections of the pancreatic head [3,4,10,32,33,34]. Recent papers comparing pancreaticoduodenectomy and distal pancreatectomy suggest that POH and PPAP differ in their clinical relevance and that the criteria proposed by Connor [1] may not be uniformly applicable to both types of pancreatectomies. Ikenaga et al. observed that, in contrast to patients developing PPAP following pancreaticoduodenectomy, the clinical course of patients undergoing distal pancreatectomy did not differ between those with and without POH [12]. In a propensity score matching analysis, Radulova–Mauersberger et al. found that POH defined according to the recent ISGPS definition was a risk factor for missing textbook outcomes following pancreaticoduodenectomy, but not distal pancreatectomy [34]. Similar results were also observed by Kühlbrey et al. [4] while Andrianello et al. described the occurrence of POH as an independent risk factor for overall complications following distal pancreatectomy [25].

The Heidelberg group observed that compared to patients without POH, severe postoperative complications were sixteen times more likely to occur in patients displaying POH greater than three times the institutional upper limit of normal on postoperative day 1. However, complication rates were only three times higher in those having POH on postoperative day 1, below the three-fold threshold [13]. This raises the question whether the required 48 h elevated amylase levels as per the ISGPS definition are merely a consequence of an initial high peak rather than having a distinct significance. In a similar vein, in our study, POH on day 1 was significantly higher in patients with PPAP in the standard group compared to the modified group.

A recently published European multicentric study identified POH as an early predictor of POPF and its severity after distal pancreatectomy [35]. Applying the ISGPS criteria, Perri et al. observed that 18% of patients developed POH; however, in half of the patients, it would just represent a non-clinically relevant biochemical finding.

Similarly, in our standard group, only 50% (12 out of 24) of the patients developing POH progressed towards PPAP. Of these 12 patients with PPAP, 4 did not develop adverse sequelae, highlighting the need to further understand the cause-and-effect relationship between POH, PPAP, and clinically relevant postoperative pancreatic fistulas.

Limitations of the present study are the retrospective study design as well as the single-center character of this study with a limited number of patients. This also includes the retrospective assessments of radiological findings, which may lead to a more meticulous diagnosis of PPAP criteria. Another potential weakness is the long study period, which covers a 13-year period with inherent inhomogeneities in treatment regimens, varying surgeons, and changes in surgical techniques. Strengths of the study are the auditable institutional database and the data granularity.

In summary, the mandatory 48 h period for POH included in the current standard definition by the ISGPS for PPAP seems to be crucial to better reflect the clinical impact of PPAP following distal pancreatectomy. Although the use of both serological and imaging criteria helps to better capture the clinical relevance of a diagnosed PPAP following distal pancreatectomy, further efforts are needed to identify a sweet spot that can better discriminate between PPAP courses that progress towards severe complications and those that do not. The ultimate aim would be to recognize a worsening clinical situation early on and eventually prevent it.

## Figures and Tables

**Table 1:** Patient demographics and surgery-related data.

Parameter	All (n = 207)
Age (years) *	63 (51–72)
Sex	
Female	103 (49.8)
Male	104 (50.2)
BMI (kg/m^2^)	26.3 (22.8–29.1)
Diabetes	39 (18.8)
Cardio-vascular disease	101 (48.8)
ASA-Classification	
1	26 (12.7)
2	124 (60.5)
3	54 (26.3)
4	1 (0.5)
Operation time (min) *	258 (205–326)
Operation technique	
Distal pancreatectomy	158 (76.3)
Pancreatic tail resection	12 (5.8)
RAMPS	37 (17.9)
Multivisceral resection	8 (3.9)
Splenectomy	119 (57.5)
PV/VMS resections	13 (6.3)
Arterial resections	10 (4.8)
Intraoperative fluid administration crystalloids (mL) *	2500 (2000–3286)
Intraoperative fluid administration colloids (mL) *	910 (500–1000)
Preoperative anticoagulation	18 (8.8)
Preoperative antiplatelet drugs	42 (20.5)
Intraoperative diuresis (mL) *	500 (300–768)

* median value (25– and 75% percentiles).

**Table 2:** Histopathological diagnoses.

Histological Diagnosis	Number of Patients (%)
Adenocarcinoma	57 (27.5)
pNEN	54 (26.1)
Benign lesions	73 (35.3)
IPMN	39 (18.8)
Other	5 (2.4)
Chronic pancreatitis	29 (14.0)
Secondary malignancies	16 (7.7)
SPN	6 (2.9)
Lymphoma	1 (0.5)

IPMN, intraductal papillary mucinous neoplasms; pNEN, pancreatic neuroendocrine neoplasms; SPN, solid pseudopapillary neoplasm.

**Table 3:** Group demographics and surgery-related data stratified by PPAP according to the ISGPS definition (standard group).

Parameter	PPAP (n = 12)	No PPAP (n = 195)	*p*-Value
Age (years) *#	60 (46–67)	63 (51–72)	0.361
Sex +			0.540
Female	7 (58.3)	96 (50.8)	
Male	5 (41.7)	99 (49.2)	
BMI (kg/m^2^) *#	26.7 (23.9–28.5)	25.6 (22.8–29.3)	0.882
Diabetes +	2 (16.7)	37 (18.9)	0.755
Cardio-vascular disease +	6 (50.0)	96 (49.2)	0.931
ASA-Classification +			0.876
1	2 (16.7)	24 (12.3)	
2	6 (50.0)	119 (61.0)	
3	4 (33.3)	51 (26.2)	
4	0 (0.0)	1 (0.5)	
Operation time (min) *#	195 (145–304)	270 (217–328)	0.850
Operation technique +			0.579
Distal pancreatectomy	7 (58.3)	151 (77.4)	
Pancreatic tail resection	1 (8.3)	11 (5.7)	
RAMPS	4 (33.3)	33 (16.9)	
Multivisceral resection +	0 (0.0)	8 (10.3)	0.410
Splenectomy +	7 (58.3)	112 (57.4)	0.452
PV/VMS resection +	0 (0.0)	13 (6.6)	0.887
Arterial resection +	1 (8.3)	9 (4.6)	0.560
Intraoperative fluid administration crystalloids (mL) *#	2607 (1880–3183)	2500 (2000–3305)	0.757
Intraoperative fluid administration colloids (mL) *#	1000 (163–1000)	660 (500–1000)	0.839
Preoperative anticoagulation +	1 (8.3)	17 (8.8)	0.955
Preoperative antiplatelet drugs +	2 (16.7)	40 (20.7)	0.735
Intraoperative diuresis (mL) *#	495 (325–848)	500 (300–768)	0.847

ASA, American Society of Anesthesiologists—Classification; BMI, Body Mass Index; PV, portal vein; RAMPS, Radical antegrade modular pancreatosplenectomy; VMS, superior mesenteric vein; * median value (25– and 75% percentiles); other values: mean (%); + Fisher’s Exact Test; # Mann–Whitney U Test.

**Table 4:** Postoperative course—stratified by PPAP according to the ISGPS definition (standard group).

Parameter	All (n = 207)	PPAP (n = 12)	Non-PPAP (n = 195)	*p*-Value
Serum amylase postoperative day 1 *#	74 (46–1735)	205 (78–1440)	41 (22–138)	0.002
POH +	24 (11.6)	12 (100)	12 (6.2)	<0.001
CR-POPF +	49 (23.7)	8 (66.7)	41 (21.0)	<0.001
PPH +	16 (7.7)	5 (41.7)	11 (5.6)	<0.001
Relaparotomy (90 days) +	29 (14.0)	4 (33.3)	25 (12.8)	0.069
Pleural effusion +	41 (21.6)	7 (58.3)	34 (17.4)	0.005
Peripancreatic fluid collection +	68 (32.9)	8 (66.7)	60 (30.8)	0.002
Perioperative transfusion +	33 (16.3)	2 (16.7)	31 (15.9)	0.562
Readmission (90 days) +	39 (19.0)	4 (33.3)	35 (17.9)	0.193
Major complications (Clavien–Dindo ≥ III) +	54 (26.1)	8 (66.7)	46 (23.6)	<0.001
LOS (days) *#	12 (8–19)	27 (11–47)	12 (8–17)	0.002
Mortality (90 days) +	5 (2.4)	0 (0.0)	5 (2.6)	0.771
Costs (€) *#	13,231 (11,119–16,293)	15,116 (10,378–51,182)	13,226 (11,129–16,285)	0.002

CR-POPF, clinical-relevant postoperative pancreatic fistula; LOS, length of stay; POH, postoperative hyperamylasemia; PPH, post-pancreatectomy hemorrhage; * median value (25– and 75% percentiles); other values: mean (%); + Fisher’s Exact Test; # Mann–Whitney U Test.

**Table 5:** Group demographics and surgery-related data stratified by PPAP according to the modified definition (modified group).

Parameter	PPAP (n = 12)	No PPAP (n = 180)	*p*-Value
Age (years) *#	57 (45–65)	64 (51–72)	0.139
Sex +			0.372
Female	15 (55.6)	88 (48.9)	
Male	12 (44.4)	92 (51.1)	
BMI (kg/m^2^) *#	26.3 (23.8–30.1)	25.7 (22.7–29.1)	0.771
Diabetes +	5 (18.5)	34 (18.9)	0.779
Cardio-Vascular disease +	13 (48.1)	89 (49.4)	0.900
ASA-Classification +			0.876
1	4 (14.8)	22 (12.2)	
2	14 (51.9)	111 (61.7)	
3	9 (33.3)	46 (25.6)	
4	0 (0.0)	1 (0.5)	
Operation time *#	252 (202–315)	234 (184–300)	0.283
Operation technique +			0.893
Distal pancreatectomy	19 (70.4)	139 (77.2)	
Pancreatic tail resections	2 (7.4)	10 (5.6)	
RAMPS	6 (22.2)	31 (17.2)	
Multivisceral resection +	0 (0.0)	8 (4.4)	0.985
Splenectomy +	7 (25.9)	112 (62.2)	0.450
PV/VMS resection +	4 (14.8)	9 (5.0)	0.027
Arterial resection +	1 (3.7)	9 (5.0)	0.872
Intraoperative fluid administration crystalloids (mL) *#	2412 (2000–3155)	2500 (2000–3310)	0.485
Intraoperative fluid administration colloids (mL) *#	1000 (500–1000)	500 (500–1000)	0.099
Preoperative anticoagulation +	2 (7.4)	16 (8.8)	0.934
Preoperative antiplatelet drugs +	2 (16.7)	3 (11.1)	0.116
Intraoperative diuresis (mL) *#	460 (315–763)	510 (300–773)	0.522

ASA, American Society of Anesthesiologists—Classification; BMI, Body Mass Index; PV, portal vein; RAMPS, Radical antegrade modular pancreatosplenectomy; VMS, superior mesenteric vein; * median value (25– and 75% percentiles); other values: mean (%); + Fisher’s Exact Test; # Mann–Whitney U Test.

**Table 6:** Postoperative course—stratified by PPAP according to the modified definition (modified group).

Parameter	All (n = 207)	PPAP (n = 27)	Non-PPAP (n = 180)	*p*-Value
Serum amylase postoperative day 1 *#	74 (46–1735)	138 (42–935)	54 (24–68)	0.008
POH +	47 (22.7)	27 (100)	29 (11.1)	<0.001
CR-POPF	49 (23.7)	12 (44.4)	37 (20.1)	0.027
PPH +	16 (7.7)	7 (25.9)	9 (5.0)	<0.001
Relaparotomy (90 days) +	29 (14.0)	6 (22.2)	23 (12.8)	0.099
Pleural effusion +	41 (21.6)	10 (37.0)	31 (17.2)	0.073
Peripancreatic fluid collection +	68 (32.9)	16 (59.3)	52 (28.9)	0.018
Perioperative transfusion +	33 (16.3)	4 (14.8)	29 (16.1)	0.632
Readmission (90 days) +	39 (19.0)	7 (25.9)	32 (17.8)	0.427
Major complications (Clavien–Dindo ≥ III) +	54 (26.1)	12 (44.4)	42 (23.3)	0.007
LOS (days) *#	12 (8–19)	14 (10–28)	12 (8–17)	0.014
Mortality +	5 (2.4)	0 (0.0)	5 (2.8)	0.533
Costs (€) *#	13,231 (11,119–16,293)	13,537 (9992–20,439)	11,229 (8996–16,163)	0.041

CR-POPF, clinical-relevant postoperative pancreatic fistula; LOS, length of stay; POH, postoperative hyperamylasemia; PPH, post-pancreatectomy hemorrhage; * median value (25– and 75% percentiles); other values: mean (%); + Fisher’s Exact Test. # Mann–Whitney U Test.

**Table 7:** Postoperative course in patients experiencing PPAP according to the two POH definitions.

Parameter	Standard Group (n = 12)	Modified Group (n = 27)	*p*-Value
Serum amylase postoperative day 1 *#	205 (78–1440)	138 (42–935)	0.026
CR-POPF +	8 (66.7)	12 (44.4)	0.053
PPH +	5 (41.7)	7 (25.9)	0.058
Relaparotomy (90 days) +	4 (33.3)	6 (22.2)	0.237
Pleural effusion +	7 (58.3)	10 (37.0)	0.002
Peripancreatic fluid collection +	8 (66.7)	16 (59.3)	0.064
Perioperative transfusion +	2 (16.7)	4 (14.8)	0.978
Readmission (90 days) +	4 (33.3)	7 (25.9)	0.587
Major complications (Clavien–Dindo ≥ III) +	8 (66.7)	12 (44.4)	0.061
LOS (days) *#	27 (11–47)	14 (10–28)	0.210
Mortality (90 days) +	0 (0.0)	0 (0.0)	n.a.
Costs (€) *#	15,116 (10,378–51,182)	13,537 (9992–20,439)	0.132

CR-POPF, clinical-relevant postoperative pancreatic fistula; LOS, length of stay; PPH, post-pancreatectomy hemorrhage; * median value (25– and 75% percentiles); other values: mean (%); + Fisher’s Exact Test; # Mann–Whitney U Test.

## Data Availability

The data presented in this study are available on request from the corresponding author due to containing patient data.

## Abbreviations

The following abbreviations are used in this manuscript:

ASA	American Society of Anesthesiologists
CD	Clavien–Dindo classification
CR-POPF	Clinically Relevant Postoperative Pancreatic Fistula
CT	Computed Tomography
IL	Illinois, USA (contextual geographic abbreviation)
IPMN	Intraductal Papillary Mucinous Neoplasm
ISGPS	International Study Group for Pancreatic Surgery
LOS	Length of Stay
n.a.	Not applicable
PORSCH	Postoperative Outcome After Resection of the Pancreas Study
POH	Postoperative Hyperamylasemia
PPAP	Post-Pancreatectomy Acute Pancreatitis
PPH	Post-Pancreatectomy Hemorrhage
PV	Portal Vein
pNEN	Pancreatic Neuroendocrine Neoplasm
RAMPS	Radical Antegrade Modular Pancreatosplenectomy
SPN	Solid Pseudopapillary Neoplasm
STROBE	Strengthening the Reporting of Observational Studies in Epidemiology
U/L	Units per Liter
VMS	Superior Mesenteric Vein
